# Parental Compliance with Preschool Vision Screening Test

**DOI:** 10.3390/jcm14010107

**Published:** 2024-12-28

**Authors:** Hilit Kerner Lavi, Tal Koval, Ilanit Trifonov, Olga Reitblat, Oriel Spierer

**Affiliations:** 1 Department of Ophthalmology, Edith Wolfson Medical Center, Holon 58100, Israel; kernerhilit@gmail.com (H.K.L.); talkovalr@gmail.com (T.K.); 2 School of Medicine, Faculty of Medical and Health Sciences, Tel Aviv University, Tel Aviv 6997801, Israel; ilanit.tri@gmail.com (I.T.); olga.reitblat@gmail.com (O.R.); 3 Department of Ophthalmology, Rabin Medical Center, Petach Tikva 4941492, Israel

**Keywords:** preschool vision screening, amblyopia, parental cooperation

## Abstract

**Objective:** To assess the barriers to parental compliance with preschool vision screening tests and the recommended follow-up eye care. **Methods:** This prospective study included children aged 3–6 years attending 46 preschools. Parents were asked for consent for their children to participate in a vision screening test. Parents whose child did not participate due to lack of parental consent and parents whose child failed the screening test were contacted by telephone and given a standardized questionnaire to identify potential barriers to compliance. **Results:** A total of 1511 children (mean age 4.76 years ± 0.76, 51.3% boys) were eligible for vision screening. Consent was given by the parents of 1295 children (85.7%). Lack of consent in children who had never been examined by an ophthalmologist was primarily due to unawareness of the screening test or other logistical reasons (117 cases, 92.1%). Of the children screened, 140 (11.1%) failed the test and 80.0% of their parents adhered to the recommended follow-up eye care. Parents who followed the screening vision test recommendations were more likely to be native language speakers (82.8% vs. 58.8% mothers and 88.9% vs. 60.0% fathers; *p* = 0.049 and 0.015, respectively). There was a higher chance of at least one parent being native-born if recommendations were followed (90.6% vs. 58.8%, *p* = 0.004). All other factors tested were insignificant. **Conclusions**: Parental consent and cooperation with vision screening test and its recommendations were high. Migrant families are more likely to face challenges in following vision screening test recommendations, underscoring the need for tailored approaches for specific populations.

## 1. Introduction

In amblyopia, the brain fails to process the visual input from one or both eyes, which, if left untreated, usually results in a reduction in visual acuity [1]. It affects 2–4% of children in developed countries and is considered the most common cause of vision loss in children [1,2]. Early diagnosis and timely treatment are of the utmost importance in restoring vision in amblyopic eyes, as amblyopia can usually be treated if diagnosed before the age of seven [2,3]. Vision screening tests at the age of three to four years can significantly reduce the risk of amblyopia from 2.39% to 0.299% [4]. In many countries, vision screening tests are performed in preschools and in first grade to detect preventable vision-threatening conditions in children, like stimulus deprivation, strabismus, and refractive errors (myopia, hyperopia, and astigmatism) [5,6,7]. Preschool vision screening is endorsed, among others, by the World Health Organization, the American Association of Pediatric Ophthalmology and Strabismus, the American Academy of Ophthalmology, and the United States Preventive Services Task Force [8,9,10].

Vision screening tests can be carried out using a photoscreener, a non-invasive, handheld, portable device that automatically and simultaneously captures vision data from both eyes. Photoscreeners do not diagnose amblyopia but instead identify risk factors associated with its development. Directly measuring visual acuity in a large number of children, many of whom may be uncooperative or unable to provide accurate responses reflecting their true vision, would have required an impractically large number of trained professionals. In contrast, a photoscreener is easy for non-professionals to use, takes approximately 10 s per test, and poses no risk to the child being screened. Using photoscreeners to detect amblyopia risk factors in young children instead of measuring visual acuity is a common practice throughout the world [7,11,12]. The device output includes refraction, pupillary size, gaze deviation, and the presence of media opacities. Sensitivity and specificity are 75–92% and 85–91%, respectively [12]. At the end of the exam, an automatic report is generated, stating whether the child passed the test or failed it, and in the latter event, further follow-up with an ophthalmologist for full eye evaluation is recommended.

Parental adherence to vision screening recommendations of follow-up eye care is variable, ranging from 25% to 84% in previous studies [5,6,7,13,14]. However, almost no data are available regarding compliance with participation in the vision screening itself. The first goal of this study was to evaluate parental compliance and cooperation, including possible barriers, with preschool vision screening tests. The second purpose was to evaluate parental compliance and possible barriers to vision screening test recommendations. The targeted children in this study were those attending first-level and second-level preschool classes (children aged 3–6 years).

## 2. Materials and Methods

### 2.1. Participants and Study Design

In this prospective study, the Ophthalmology Department at the E. Wolfson Medical Center collaborated with the Municipality of the city of Holon, Israel, to conduct vision screening tests in preschools from September to October 2022. The study received approval from the E. Wolfson Medical Center’s Internal Review Board and adhered to the principles of the Declaration of Helsinki. Two weeks before the screening, the city’s Department of Education informed parents about the upcoming vision screenings by sending letters to their homes. These letters provided information about amblyopia, the importance of early detection of risk factors, and details about the screening process. Parents were encouraged to explore additional data on the Ministry of Health website through a provided link. Consent forms were included for parents to sign and return to the preschool teachers. Trained volunteer examiners conducted vision screening tests using a photoscreener in 46 preschools. Upon completion, parents received a report indicating whether their child had passed or failed the screening test, along with recommendations for further evaluation by an ophthalmologist if needed.

Parents whose children did not participate in the vision screening test due to a lack of parental consent were contacted by telephone and asked to complete a standardized questionnaire to identify potential reasons for non-participation and collect additional demographic information (Supplementary Material S1). For children who failed the screening, their parents were contacted four weeks later to determine whether they had arranged a follow-up eye exam with an ophthalmologist. If no follow-up was conducted, parents were asked about potential barriers to seeking care, and relevant demographic data were collected (Supplementary Material S2).

### 2.2. Vision Screening Test

The vision screening was performed using the Vision Screener plusoptiX S09 (Plusoptix Gmb, Nuernberg, Germany), which uses an infrared video to measure binocular spherical equivalents, astigmatism, asymmetry of the corneal reflexes, and pupil size in real time. With this vision screener, both eyes are measured simultaneously from a distance of 1.2 m from the face of the child. A warble sound is used to draw the child’s attention to the fixation target on the camera. It has a spherical and cylindrical range of −7.0 to +5.0 diopters (D) in increments of 0.25 D. The screening result “Pass” or “Refer” is based on the referral criteria set [15].

### 2.3. Volunteer Training

The non-professionals volunteer training program, overseen by the Lions Club Israel Commission, included a 3.5 h seminar that covered the fundamentals of amblyopia, guidance on organizing screenings, operation of the Vision Screener Plusoptix S09, and methods for identifying readable versus unreadable photographs. Participants were provided with Supplementary Materials, including the photoscreener manual and screening forms. After completing the theoretical component, trainees participated in hands-on simulations of supervised screenings and underwent a certification test. Those who successfully passed were certified to perform vision screenings in preschool settings. In this study, the screenings were additionally supervised by an ophthalmologist (H.K.L.) and a medical student (I.T.), who were also trained specifically for this purpose.

### 2.4. Statistical Analysis

SPSS version 28 (IBM, Chicago, IL, USA) was used for statistical analyses. Descriptive statistics are presented as a mean ± standard deviation (SD) along with the corresponding median and range for continuous variables. Categorical values are summarized with their relative percentage.

Continuous variables were compared using the non-parametric Mann–Whitney U test, and Pearson’s chi-square test or Fisher’s exact test was used, as appropriate, to compare patients’ categorical variables.

A multiple logistic regression analysis was conducted separately for two groups: those without parental consent and those children who failed the test and were referred for follow-up with an ophthalmologist. The analysis included potential predictive factors such as the child’s age, gender, general health condition, and family history of eye conditions. It also considered parental factors including age, country of birth, native language, educational level, and the lack of private medical insurance coverage.

All statistical analyses performed were two-sided and statistical significance was set at a *p*-value of 0.05.

## 3. Results

**Figure 1:**
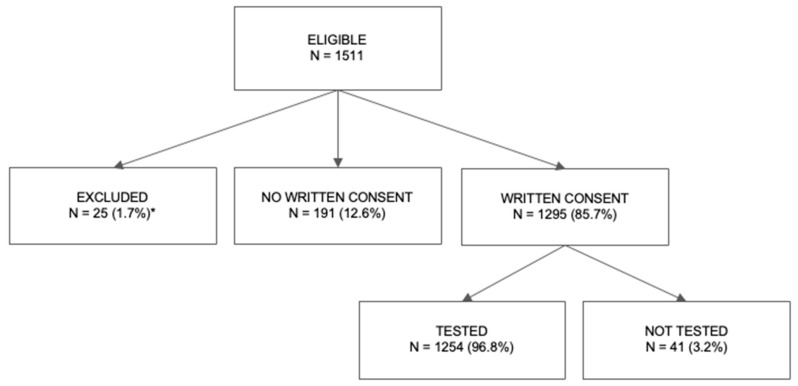
Parental consent and children’s participation in vision screening test. * Exclusion was due to inaccuracies in the council’s registry.

One thousand five hundred and eleven children attending 46 public preschools were eligible for vision screening at the time of the study. The mean age was 4.76 ± 0.76 years (range 3–6 years) and 51.3% were boys. Parents of 1295 children gave their written consent for their child to participate in the screening test, and a total of 1254 (96.8%) children were eventually tested (the remaining were absent on the day of the screening test or uncooperative during the test, Figure 1).

### 3.1. Children Who Did Not Undergo Vision Screening Test Due to Lack of Parental Consent

The parents of 191 children with no parental written consent were contacted by telephone (Table 1). Fifty-one (26.7%) of them declared that their children had recently undergone an eye examination or were already under regular ophthalmic follow-up, and therefore found the screening unnecessary. In 13 (6.8%) cases, the parents could not be reached by telephone. The remaining children (127, 66.5%) were divided into two groups: Group 1 included children whose parents seemed to understand the importance of vision screening, but for various reasons, mostly unawareness of the test or logistical reasons did not provide their written consent. Group 2 included all cases of intentional refusal, including parents who were categorically against all screening tests and those who believed their child’s vision was adequate and thus no examination was needed (Table 1).

### 3.2. Children Who Underwent Vision Screening Test

Of the 1254 children who underwent vision screening, 1114 (88.8%) children passed the test, and 140 (11.2%) failed it and were referred to an ophthalmologist. Failure was due to astigmatism in 79 children (56.4%), anisometropia in 32 (22.8%), myopia in 17 (12.1%), asymmetric corneal reflex in 15 (10.7%), hyperopia in 13 (9.2%), and anisocoria in 8 (5.7%) children (in some cases failure was due to more than one cause).

One hundred forty parents of the referred children were contacted four weeks after the screening test. Forty-two of the children were found to be under routine ophthalmological follow-up. Thirteen of the children’s parents could not be reached by phone. From the remaining 85 parents, 68 (80.0%, Group A) followed the recommendation and scheduled an appointment with an ophthalmologist, and 17 (20.0%, Group B) parents had not yet scheduled an appointment or did not intend to (Table 3).

Group A parents were more likely to be native language speakers as compared to Group B (82.8% vs. 58.8% of the mothers, and 88.9% vs. 60% of the fathers, *p* = 0.049 and 0.01, respectively). There was a higher chance of at least one parent being native-born in Group A than in Group B (90.6% vs. 58.8%, *p* = 0.004). Other parameters were similar between the two groups (Table 4).

## 4. Discussion

In this study, we observed a high level of parental consent and cooperation with vision screening tests and with their recommendations (85.7% and 80.0% of the parents, respectively). However, we also found that migrant families were more likely to encounter difficulties in adhering to vision screening test recommendations, as shown by the significantly higher likelihood of at least one parent being native-born among families who followed the recommendations (90.6% vs. 58.8%, *p* = 0.004).

Visual impairment caused by amblyopia can be severe and long-lasting if not diagnosed and treated promptly. It is estimated that 175.2 million people worldwide will be amblyopic by 2030, increasing to 221.9 million by 2040 [16]. These staggering projections should urge decision-makers to implement more efficient amblyopia screening and treatment strategies.

This study aimed to evaluate parental compliance with vision screening testing for their children and identify barriers to follow-up care after the initial screening. To the best of our knowledge, this is the first study to assess parental cooperation with preschool vision screening, which proved to be high, with 85.7% of the parents cooperating. We believe this rate could have been even higher since most parents who did not consent initially acknowledged the importance of the vision screening upon later questioning. However, they failed to provide consent mainly due to a lack of awareness of the test. Intentional refusal was rare, occurring in only 10 cases. There were no significant demographic differences between those who gave consent and those who did not, including factors such as the child’s age or gender, parental marital status, education level, migrant background, and the lack of additional private medical insurance coverage (suggestive of low family income). Communication with parents in this study was limited to written letters sent home with the children. Utilizing multiple communication channels, such as phone calls or digital media, could possibly enhance parental engagement and increase participation rates. Active interventions can increase parental cooperation immensely in cases where the child failed the screening test. One such program is the one designed by Pizzi et al. [17]. In their interventional program, a social worker contacted parents to address barriers and assist with scheduling follow-up appointments. As a result, adherence to follow-up increased from less than 5% to 59.2% [17]. Similar programs can be designed not only to improve adherence to follow-up but also to boost the initial participation rate in the vision screening test itself. School staff, such as teachers and teachers’ aides, could play an active role in this process, and digitalizing consent forms could make them more accessible. Another suggestion is to eliminate the need for explicit consent altogether, and to notify parents about the screening test and only require them to inform the school if they object to the test. Concerns about potential violations of children’s rights can be addressed by asking parents to complete a health declaration form at the beginning of the year, which includes their consent for vaccinations and screening tests. During the year, parents who wish to exempt their child from a screening must notify the relevant health authority or school. If no opt-out notification is provided, the child will likely participate in the screening. This approach could further simplify the process and increase participation.

Previous studies have reported varying rates of parental adherence to vision screening recommendations, ranging from 25% to 84% [5,6,7,14,18,19,20]. Our study found a follow-up rate of 80.0%, which is higher than most previous reports. Understanding the factors influencing parental compliance is essential to increase follow-up rates. Pizzi et al. categorized the barriers for follow-up into three factors: 1. predisposing parental factors (described as lack of awareness, level of perceived importance, conflict of commitment, and lack of communication means); 2. system factors (described as lack of referrals, scheduling difficulties and lack of access to transportation); and 3. financial factors (lack of insurance, health care, and transportation payment difficulties) [17]. They found that the main barriers in their study were parental awareness, perceived importance of the recommendations, conflict of prior commitments, and lack of communication means. Kemper et al. found that barriers to follow-up in a US population were lack of insurance coverage, inconvenience of follow-up, and lack of knowledge about the benefits of early intervention [14]. They also found that minority children and those with low family income were less likely to continue follow-up. In a German study, Schuster et al. found that adherence to a recommendation of a vision screening test was lower in children from low socioeconomic status and in children with a migration background [19]. Ravindran et al. found in an Indian population that low levels of education, low income, and geographic distance factors were the main barriers to parents’ adherence to test recommendations [21]. In contrast, in an Israeli study held in a district with an extremely variable ethnic and socioeconomic population, adherence to follow-up was not related to ethnicity or socioeconomic status [22]. Similarly, Su et al. found in a US population no correlation to the child’s ethnicity, health insurance status, education or employment status, and household income [13]. In Israel, the healthcare system is organized such that from birth all residents are medically covered by one of the four public health insurance agencies. In addition, from birth until the age of six, the free-of-charge Family Care Center is responsible for monitoring growth and development, providing education and guidance, and administering immunizations. Public medical insurance in Israel is free and mandatory for all residents regardless of their citizenship status, allowing easier accessibility to general ophthalmologists and pediatric ophthalmologists, usually within several weeks or less. This probably contributed to the overall high follow-up rate found in our study and explains why lack of private insurance coverage (as a possible marker for socioeconomic status) was not found to be a barrier for further follow-up. We found no correlation between adherence to follow-up and parental marital, education level, or age. The only barrier to follow-up with an ophthalmologist was migrant status. Our results may indicate that migrants’ families may encounter difficulties in scheduling a follow-up doctor appointment due to language barriers and not due to financial restrictions and may benefit from individual targeting following the vision screening test to improve follow-up rate.

A limitation of our study is that the follow-up data were collected via telephone parent surveys rather than a review of medical records, which might affect the accuracy of the reported follow-up care. An additional limitation of our study, as with all studies involving photoscreeners, is that these devices do not detect amblyopia directly but instead identify conditions that can lead to amblyopia. As a result, there is the potential for a higher referral rate. Another limitation is the potential underdiagnosis of refractive errors. The low prevalence of hyperopia detected in our study can be attributed to the screening technique used, which does not involve the application of cycloplegic eyedrops. While the primary benefit of photoscreening is that it can be performed by semi-skilled lay volunteers, they are unable to administer these drops. In Israel, comprehensive eye examinations by an ophthalmologist are recommended for all children at ages 1, 3, and 6 years. However, these exams are not mandatory and cannot be conducted in schools. Consequently, most children do not undergo these examinations, emphasizing the need for vision screening tests as was undertaken in the current study.

In conclusion, parents’ agreement to cooperate with the preschool vision screening test was high in this study. Most parents who did not initially consent had done so because they were unaware of the test being held. In addition, most parents followed the recommendation and scheduled an appointment with an ophthalmologist. Migrant families have lower adherence to follow-up rates. This finding highlights the need for specific strategies to support these populations.

## Figures and Tables

**Table 1:** Reasons for lack of parental written consent for the vision screening test for 127 children.

Parents’ Response	N (%)
**Group 1**	**117 (92.1)**
I was unaware that the test was being conducted or did not receive the consent form for signing	85 (66.9)
I have signed the consent form, but the examiners did not receive it	21 (16.5)
I prefer to be present during my child’s test	6 (4.7)
I would rather have the test conducted in a different setting and scheduled an ophthalmologist examination	5 (3.9)
**Group 2**	**10 (7.9)**
I decline all screening tests	5 (3.9)
I do not believe my child has any ocular or vision issues; therefore, I see no need for the screening test	4 (3.2)
I believe that my child is too young for a vision screening test	1 (0.8)

No difference was found between Groups 1 and 2 in demographics, general health condition, and the availability of private medical insurance (*p* > 0.05 for all, Table 2).

**Table 2:** Comparison of demographic parameters of the two groups with no written consent: Group 1 (parents who seem to understand the importance of the screening test) and Group 2 (parents who declined the screening test intentionally).

Parameter	Group 1 117 (92.1)	Group 2 10 (7.9)	*p*-Value
Girls (%)	59 (50.4)	5 (50)	0.98
Age, years (SD)	4.7 (0.8)	4.5 (0.8)	0.41
General good health condition (%)	91 (95.8)	6 (85.7)	0.30
Private medical insurance (%)	88 (93.6)	7 (87.5)	0.45
Known ocular diseases in the family (Amblyopia or strabismus, %)	15 (15.6)	0 (0)	0.60
Mother’s age, years (SD)	36.3 (4.3)	37.6 (4.9)	0.65
Father’s age, years (SD)	38.9 (5.4)	38.8 (2.7)	0.92
Mother is native-born (%)	68 (74.7)	6 (100)	0.33
Father is native-born (%)	64 (71.9)	5 (100)	0.32
At least one parent is native-born (%)	72 (77.4)	6 (100)	0.34
Mother is a native language speaker (%)	77 (81.9)	6 (85.7)	1.0
Father is a native language speaker (%)	70 (79.5)	5 (83.3)	1.0
Parental status, married (%)	59 (95.2)	13 (86.7)	0.25
Mother’s education (%)			0.23
Primary/secondary education	34 (39.5)	4 (66.7)	
Higher education	52 (60.5)	3 (33.3)	
Father’s education (%)			1.0
Primary/secondary education	41 (50.6)	3 (50.0)	
Higher education	40 (49.4)	3 (50.0)	

SD, standard deviation.

**Table 3:** Parents’ response of 85 children who failed the vision screening test and were referred to an ophthalmologist.

Parents’ Response	N (%)
**Group A: Followed the recommendation**	**68 (80.0)**
I scheduled an appointment, but the appointment date has not yet arrived	22 (25.9)
My child was examined and needs further follow-up	19 (22.4)
My child was examined, and glasses were prescribed	15 (17.6)
My child was examined, and the exam was normal	12 (14.1)
**Group B: Did not follow the recommendation**	**17 (20.0)**
I didn’t make an appointment yet, but intend to	10 (11.8)
I don’t think my child has an eye problem	5 (5.9)
I was unsure about how to make an appointment	2 (2.4)

**Table 4:** Comparison of demographic parameters of the two groups who failed the vision screening test and were referred to an ophthalmologist: Group A (followed the recommendation) and Group B (did not follow the recommendation).

Parameter	Group A 68 (80.0)	Group B 17 (20.0)	*p*-Value
Boys (%)	39 (57.4)	8 (47.1)	0.59
Age, years (SD)	4.65 (0.78)	4.8 (0.7)	0.56
General good health condition (%)	59 (90.8)	15 (88.2)	0.67
Private medical insurance (%)	50 (84.4)	12 (75.0)	0.46
Known ocular diseases in the family (Amblyopia or strabismus, %)	10 (15.6)	0 (0)	0.19
Mother’s age, years (SD)	37.3 (6.3)	37.4 (6.2)	0.42
Father’s age, years (SD)	40.2 (6.8)	39.8 (8.0)	0.84
Mother is native-born (%)	46 (71.9)	10 (58.8)	0.30
Father is native-born (%)	53 (84.1)	9 (60.0)	0.07
At least one parent is native-born (%)	58 (90.6)	10 (58.8)	<0.01
Mother is a native language speaker (%)	53 (82.8)	10 (58.8)	0.49
Father is a native language speaker (%)	56 (88.9)	9 (60.0)	0.01
Parental status, married (%)	59 (95.2)	13 (86.7)	0.25
Mother’s education (%)			0.40
Primary/secondary education	28 (45.2)	5 (33.3)	
Higher education	34 (54.8)	10 (66.7)	
Father’s education (%)			0.55
Primary/secondary education	41 (66.1)	8 (57.1)	
Higher education	21 (33.9)	6 (42.9)	

## Data Availability

The raw data supporting the conclusions of this article will be made available by the authors on request.

## Supplementary Materials

The following supporting information can be downloaded at: https://www.mdpi.com/article/10.3390/jcm14010107/s1, Supplementary Material S1: Questionnaire following vision screening: For parents who did not provide consent for the screening test. Supplementary Material S2: Questionnaire following vision screening: For parents of a child who failed the test and was referred to an ophthalmologist.
