# Supplementary material for: Parental Compliance with Preschool Vision Screening Test
Source: J Clin Med. 2024 Dec 28;14(1):107. doi: 10.3390/jcm14010107 (PMC11721629; doi:10.3390/jcm14010107)
Supplement: Supplementary file 1 [file jcm-14-00107-s001.zip › Supplementary Material S1.pdf]

Supplementary Material S1: Questionnaire following vision screening:

For parents who did not provide consent for the screening test

Date: ________________

Answering the questionnaire: Father/ Mother/ Guardian (specify: ______________)

- Child’s details: Name: ____________

  Gender: F / M

  Age: ___________________

  Preschool: _______________

  Is the child healthy?

  1. Yes.
  2. No. Specify: _______________

- Is the child insured by a private medical insurance: Yes / No
- Are there ocular (eye -related) problems in the family? Specifically, strabismus or amblyopia (crossed eyes or “lazy eye”)

  1. No
  2. Yes. Specify: _______________

- Parent’s details:

  Father's country of origin: ________

  Mother's country of origin: _______

  Father's native language: Hebrew /Russian /Arabic/Amharic/ other (specify: ____)

  Mother's native language: Hebrew /Russian /Arabic /Amharic/ other (specify: ____)

  Parental status: Married / divorced/ Single parent/ widowed. Other Specify: _______________

  Father's education: primary/ secondary / Higher education

  Mother's education: primary/ secondary / Higher education

  Father's age: _______

  Mother's age: ______

Why did you not sign the consent form?

Multiple choices answer (an open answer is also possible):

1. I was unaware of the test/ I did not receive the consent form.

2. No need, the child was recently examined by an ophthalmologist, and everything was found to be normal.

3. There is no need, the child has a known ocular problem and is regularly monitored by an ophthalmologist.

4. I don't think the child has an eye problem and everything is fine.

5. My child is too young to be tested.

6. My child is too young to wear glasses.

8. I can’t afford to buy glasses if needed

9. I wanted to be present at the test with my child

10. I object all screening tests

11. I signed the form, but the document did not reach the examiners

12. I prefer to perform an independent ophthalmology exam

13. Other reason: ____
