# Supplementary material for: Parental Compliance with Preschool Vision Screening Test
Source: J Clin Med. 2024 Dec 28;14(1):107. doi: 10.3390/jcm14010107 (PMC11721629; doi:10.3390/jcm14010107)
Supplement: Supplementary file 1 [file jcm-14-00107-s001.zip › Supplementary Material S2.pdf]

Supplementary Material S2: Questionnaire following vision screening:

For parents of a child who failed the test and referred to an ophthalmologist

Date: ________________

Answering the questionnaire: Father/ Mother/ Guardian (specify: ______________)

- Child's details: Name: ____________

  Gender: F / M

  Age: ___________________

  Preschool: _______________

  Is the child healthy?

  1. Yes.
  2. No. Specify: _______________

- Is the child insured by a private medical insurance: Yes / No
- Are there ocular (eye -related) problems in the family? Specifically, strabismus or amblyopia (crossed eyes or "lazy eye")

  1. No

  2.Yes. Specify: _______________

- Parent's details:

  Father's country of origin: ________

  Mother's country of origin: _______

  Father's native language: Hebrew /Russian /Arabic/Amharic/ other (specify: ____)

  Mother's native language: Hebrew /Russian /Arabic /Amharic/ other (specify: ____)

  Parental status: Married / divorced/ Single parent/ widowed. Other Specify: _______________

  Father's education: primary/ secondary / Higher education

  Mother's education: primary/ secondary / Higher education

  Father's age: _______

  Mother's age: ______

Did you continue with follow up as advised after the test?

Multiple choice answer (an open answer is also possible):

1. Yes. My child was examined by a **pediatric ophthalmologist** –
   Specify: Name of ophthalmologist: _____________
   Findings on examination: No problem/ Needs treatment or glasses/ Needs follow-up. Other: ______________________
2. Yes. My child was examined by a **general ophthalmologist** –
   Specify: Name of ophthalmologist: _____________
   Findings on examination: No problem/ Needs treatment or glasses/ Needs follow-up. Other: ______________________
3. Yes. My child was examined by an **optometrist**–
   Specify: Name of optometrist _____________
   Findings on examination: No problem/ Needs treatment or glasses/ Needs follow-up. Other: ______________________
4. Yes. My child was examined by an **orthoptist**–
   Specify: Name of orthoptist _____________
   Findings on examination: No problem/ Needs treatment or glasses/ Needs follow-up. Other: ______________________

5. I Made an appointment, but the appointment date has not yet arrived
6. I did not make an appointment yet, but I intend to make one.
7. No need, the child was recently examined by an ophthalmologist, and everything was normal.
8. There is no need, the child has a known eye problem and is regularly monitored by an ophthalmologist.
9. I don't think my child has an eye problem; everything is fine.
10. My child is too young to be tested.
11. My child is too young to wear glasses
12. I did not receive the test results and did not know how to make an appointment.
13. Other reason: _________________
